# Four‐year long‐term drug survival of dupilumab analyzed by treatment period in patients with moderate to severe atopic dermatitis: A real‐world retrospective study

**DOI:** 10.1111/1346-8138.17017

**Published:** 2023-11-10

**Authors:** Da‐Hyun Kang, Soon‐Hyo Kwon, Bark‐Lynn Lew

**Affiliations:** ^1^ Department of Dermatology Kyung Hee University Hospital at Gang‐dong, Kyung Hee University School of Medicine Seoul Korea

Dear Editor,

Dupilumab is a human monoclonal antibody that inhibits the signaling of interleukin (IL)‐4 and IL‐13, which are key inflammatory cytokines of atopic dermatitis (AD). Although dupilumab has been proven to be effective for the long‐term treatment of AD,^1^, ^2^ several patients discontinue it for various reasons. This study aimed to investigate the drug survival of dupilumab and reasons for discontinuation analyzed by treatment period.

This retrospective analysis included patients aged ≥18 years with moderate to severe AD treated with dupilumab from March 2019 to April 2023. Demographics, laboratory findings, and AD severity scored by the Eczema Area and Severity Index (EASI) were collected. For patients who discontinued dupilumab therapy, data on the duration of dupilumab treatment and cause of discontinuation were also collected. The efficacy of dupilumab was evaluated at weeks 16 and 52 using EASI and EASI‐75, and ‐90 (an improvement of at least 75% and 90% from the baseline) and all adverse events were recorded.

A total of 102 patients were included in the study (Table 1). Among them, 20 (19.6%) discontinued dupilumab after a mean of 29.9 weeks. The overall probability of dupilumab survival at 4 years was 80.4%. In the dupilumab continuation group, the proportion of females was significantly lower and the mean total IgE level was significantly higher than in the discontinuation group. There was no significant difference in others.

**TABLE 1:** Baseline demographics, efficacy and adverse events after dupilumab treatment between the dupilumab continuation and dupilumab discontinuation group and reasons for discontinuation analyzed by treatment period.

a				
	All patients (*n* = 102)	Dupilumab continuation (*n* = 82)	Dupilumab discontinuation (*n* = 20)	*p*‐Value
Age, mean ± SD, years	31.41 ± 12.595	31.90 ± 13.10	29.40 ± 10.30	0.200
Sex, *n* (%)				0.003*
Male,	73 (71.6)	64 (78.0)	9 (45.0)	–
Female	29 (28.4)	18 (22.0)	11 (55.0)	–
Disease onset, mean ± SD, years	15.57 ± 12.511	16.10 ± 13.08	13.40 ± 9.84	0.193
Infancy/childhood onset AD, *n* (%)	74 (72.5)	58 (70.7)	16 (80.0)	0.578
Adult‐onset AD, *n* (%)	28 (27.5)	24 (29.3)	4 (20.0)	–
Allergy comorbidities, *n* (%)	19 (18.6)	16 (19.5)	3 (15.0)	0.459
Allergic rhinitis, *n* (%)	12 (11.8)	9 (11.0)	3 (15.0)	0.431
Allergic conjunctivitis, *n* (%)	2 (2.0)	2 (2.4)	0 (0.0)	0.645
Asthma, *n* (%)	6 (5.9)	5 (6.1)	1 (5.0)	0.665
Inhalant/food allergy, *n* (%)	8 (7.8)	8 (9.8)	0 (0.0)	0.163
Laboratory findings				
Total IgE, IU/mL (range)	4392.65 ± 7109.06	5041.62 ± 7759.79	1830.92 ± 2210.73	0.020*
Eosinophil % (range)	6.54 ± 5.07	6.58 ± 5.06	6.38 ± 5.23	0.872
Eosinophil count (range)	495.12 ± 418.55	499.64 ± 419.34	476.56 ± 425.61	0.709
Previous treatments				
Systemic corticosteroid, *n* (%)	73 (71.6)	61 (74.4)	12 (60.0)	0.159
Cyclosporine, *n* (%)	97 (95.1)	79 (96.3)	18 (90.0)	0.252
Topical steroid, *n* (%)	67 (65.7)	52 (63.4)	15 (75.0)	0.240
Topical calcineurin inhibitor, *n* (%)	87 (85.3)	70 (85.4)	17 (85.0)	0.602
Initial EASI	26.119 ± 7.99	26.202 ± 7.95	26.010 ± 8.31	0.641
Week 16 EASI	5.515 ± 3.08 (*n* = 90)	5.670 ± 3.23 (*n* = 76)	4.679 ± 2.02 (*n* = 14)	0.286
EASI 75, *n* (%)	59 (65.6)	49 (64.5)	10 (71.4)	0.431
EASI 90, *n* (%)	8 (8.9)	6 (7.9)	2 (14.3)	0.362
Week 52 EASI	3.098 ± 1.85 (*n* = 59)	3.040 ± 1.86 (*n* = 55)	3.900 ± 1.04 (*n* = 4)	0.287
EASI 75, *n* (%)	54 (91.5)	51 (92.7)	3 (75.0)	0.305
EASI 90, *n* (%)	26 (44.1)	25 (45.5)	1 (25.0)	0.402
Adverse events, *n* (%)	36 (35.3)	27 (33.0)	9 (45.0)	0.574
Head and neck erythema, *n* (%)	19 (18.6)	15 (18.3)	4 (20.0)	0.541
Conjunctivitis, *n* (%)	11 (10.8)	8 (9.8)	3 (15.0)	0.369
Injection site reaction, *n* (%)	4 (3.9)	3 (3.7)	1 (5.0)	0.588
Alopecia areata, *n* (%)	2 (2.0)	1 (1.2)	1 (5.0)	0.355

b. Reasons of dupilumab discontinuation analyzed by treatment period				
	Total (*n* = 25)	Within 6 months (*n* = 12)	6 month – 1 year (*n* = 5)	After 1 year (*n* = 8)
Loss of efficacy, *n* (%)	10 (40.0)	6 (50)	2 (40.0)	2 (25.0)
Adverse events, *n* (%)	5 (20.0)	1 (8.3)	1 (20.0)	3 (37.5)
High cost, *n* (%)	4 (16.0)	3 (25.0)	1 (20.0)	0
Discomfort with short treatment interval, *n* (%)	3 (12.0)	0	1 (20.0)	2 (25.0)
Pregnancy, *n* (%)	2 (8.0)	1 (8.3)	0	1 (12.5)
Clinical remission, *n* (%)	1 (4.0)	1 (8.3)	0	0

All values after ± is mean. SDAD, atopic dermatitis; EASI, eczema area and severity index; EASI 75, an improvement of at least 75% from the baseline EASI; EASI 90, an improvement of at least 90% from the baseline EASI.

* *p* < 0.05.

In the dupilumab discontinuation group, duplicatable reasons (*n* = 25) for withdrawing were primary loss of efficacy (40.0%), adverse events (20.0%), high cost (16.0%), discomfort with short interval between treatments (12.0%), pregnancy (8.0%), and clinical remission (4.0%). According to the time from dupilumab initiation to discontinuation, in cases of within 6 months, most patients discontinued due to dissatisfaction with the loss of efficacy (50.0%) or burdened with high costs (25.0%). However, in cases of >1 year, the ratio of patients who discontinued due to adverse events (37.5%) or discomfort with the short interval between treatments (25.0%) become higher.

Our study provides a long‐term survival analysis of dupilumab and the overall probability at 4 years was 80.4%. According to previous studies,^3^, ^4^ most 2‐year survival rates are in the late 80% range. Recently, Pezzolo et al.^5^ reported a 4‐year long‐term drug survival of approximately 76.4% for dupilumab, and our result was slightly higher.

The leading cause of discontinuation was inefficacy. However, the mean EASI scores at weeks 16 and 52 were not significantly different between the two groups. This shows that discontinuation due to inefficacy resulted from individual subjective satisfaction, mostly dissatisfaction with paradoxical face and neck flare. Strengths from the current study is that we analyzed the reasons of discontinuation by treatment period. Our study confirmed that the longer the dupilumab treatment period, the higher the rate of discontinuation due to discomfort with short interval between treatments requiring a visit the hospital every 2 weeks, or adverse events such as paradoxical facial erythema. The limitations of our study are its retrospective design, which is dependent on the quality of the medical records, and the small sample size.

## CONFLICT OF INTEREST STATEMENT

None declared.
